# A Case of Intraparenchymal Hepatic Hemorrhage Due to Ticagrelor Loading Treatment in an Elderly Patient With Acute Coronary Syndrome

**DOI:** 10.7759/cureus.13318

**Published:** 2021-02-12

**Authors:** Cagdas Kaynak, Muzaffer Aslan

**Affiliations:** 1 Cardiology, Siirt Education Research Hospital, Siirt, TUR

**Keywords:** ticagrelor, de-escalation, acute coronary syndrome, elderly, intraparenchymal hemorrhage

## Abstract

Ticagrelor is a potent P2Y12 inhibitor that is increasingly used in acute coronary syndrome. Although drug-related intrahepatic hemorrhages have been reported with anticoagulant drugs, no case of intrahepatic hemorrhage due to ticagrelor has been encountered in the literature. With this case, we tried to explain that potent antiaggregant drugs may cause this rare bleeding complication. Although ticagrelor is used for rapid antiaggregation effect in acute coronary syndrome, de-escalation to clopidogrel treatment in elderly patients during the hospitalization period is a more reasonable option.

## Introduction

Ticagrelor is a direct-acting, reversible P2Y12 inhibitor and is recommended for use in patients with the acute coronary syndrome (ACS) in line with the guidelines due to its strong antiplatelet activity and rapid onset of action and pleiotropic effects on the endothelium compared to conventional antiaggregant drugs. Despite this strong antiaggregant efficacy advantage, drug-induced dyspnea and drug-related bleeding complications are seen as the most important disadvantages of drug use [1].

Intraparenchymal hepatic hemorrhages are generally seen secondary to severe trauma; on the other hand, rarely can they also be seen due to used anticoagulant treatment, pregnancy and iatrogenic reasons [2-4]. In the literature review, although diffuse alveolar hemorrhage developed in the lung secondary to ticagrelor has been observed, no intraparenchymal hepatic hemorrhage case due to ticagrelor use was found without trauma [5]. With this case, we aimed to present this rare bleeding complication due to the use of ticagrelor and to discuss the early de-escalation process to clopidogrel therapy in elderly patients.

## Case presentation

**Figure 1:**
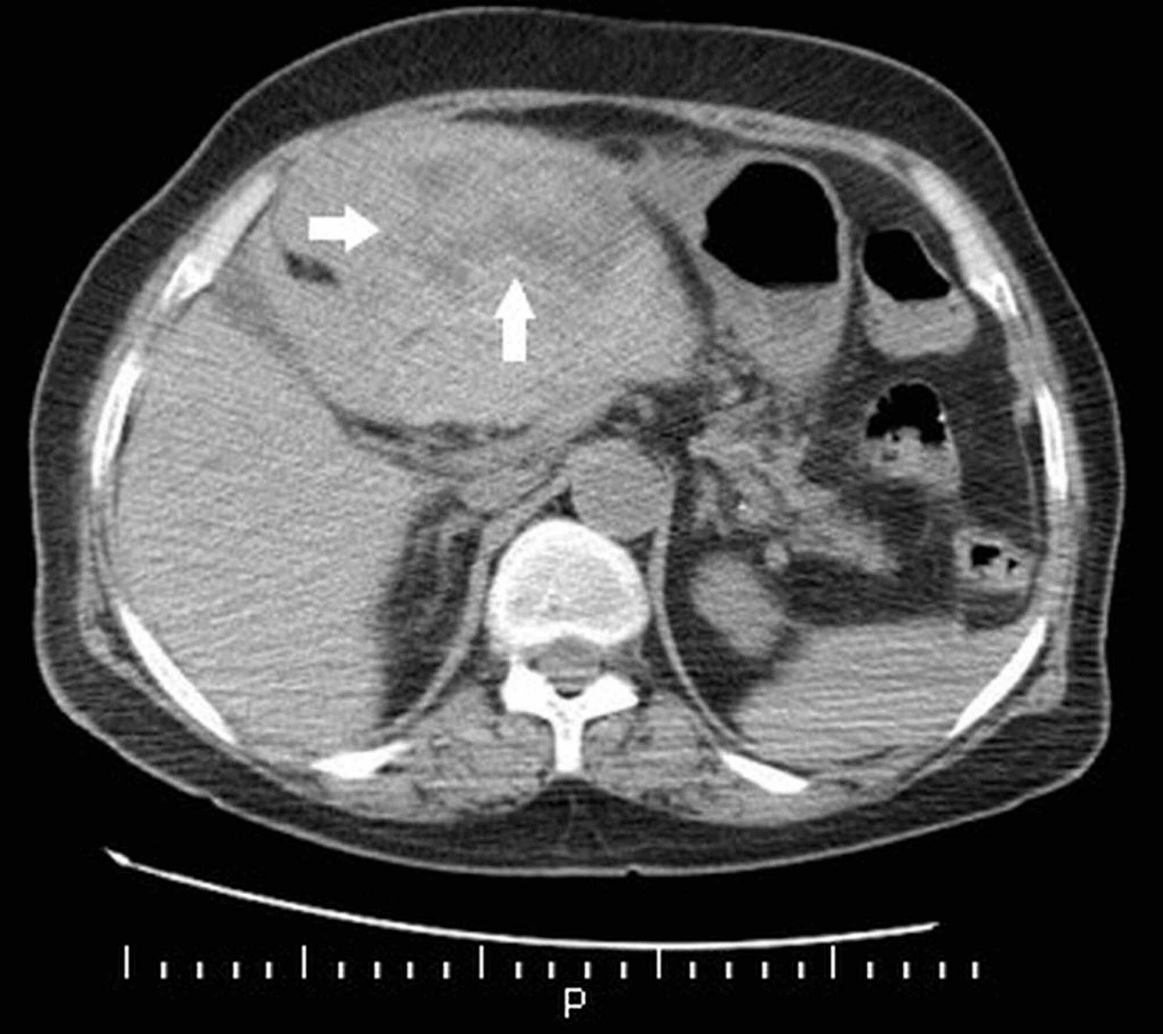
Abdomen computed tomography image: intraparenchymal hemorrhage-hematoma area in the left liver lobe with heterogeneous character around 8 cm (white arrows)

A 78-year-old female patient was admitted to the emergency department with acute anterior ST-segment elevation myocardial infarction (MI). On arrival, the patient’s electrocardiography (ECG) was in normal sinus rhythm and V1-V6 anterior leads ST segments were elevated. The patient was in a hemodynamically hypotensive cardiogenic shock state. Ejection fraction (EF) on echocardiography 30% and anterior apical segments were hypokinetic. The general condition of the patient was poor; therefore, she was intubated, subsequently, by nasogastric route 300 mg of acetylsalicylic acid and 180 mg of ticagrelor, subcutaneous low molecular weight heparin and intravenous positive inotropic support were administered. As a result of emergency coronary angiography, after successful percutaneous transluminal coronary angioplasty (PTCA) for a total occluded lesion in the left anterior descending (LAD) artery with a 2.0*12 mm balloon, then 3.0*23 mm Resolute Integrity drug-coated stent (Medtronic Inc., Minneapolis, MN, USA) was implanted. The patient was extubated on the first day of follow-up. The patient whose hemodynamics recovered was transferred to the service during follow-up. While discharge was planned in the service on the third day, it was observed that hemoglobin value decreased from 12 g/dL to 6.1 g/dL (reference value: 12-16 g/dL) in routine blood tests. The patient did not have any symptoms. In the biochemical analysis, C-reactive protein (CRP): 175.2 mg/L (reference value: 0-10 mg/L) increased significantly. Procalcitonin: 0.21 (reference value: <0.12) slightly increased but not significantly high. Liver function tests - aspartate aminotransferase (AST): 45 U/L (reference value: 0-34 U/L) and alanine aminotransferase (ALT): 139 U/L (reference value: 10-49 U/L) were minimally increased. Total bilirubin: 0.9 mg/dL (reference value: 0.2-1.2 mg/dL), platelet count (Plt): 203,000/µL (reference value: 130,000-400,000/µL), prothrombin time (PTZ): 12.7 sec (reference value: 12-17 sec) and international normalized ratio (INR): 0.99 (reference value: 0.8-1.3) levels were normal. In the follow-up, non-contrast abdomen computed tomography (CT) imaging was performed and this showed that there were intraparenchymal hemorrhage-hematoma areas in the left liver lobe with heterogeneous character around 8 cm (Figure 1).

**Figure 2:**
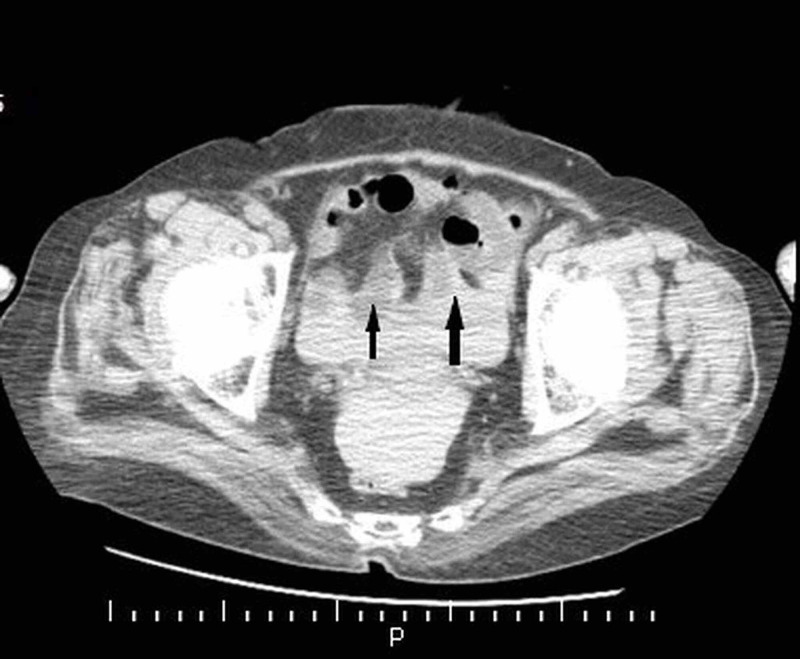
Abdomen computed tomography image: high-density fluid image around intra-abdominal organs compatible with intra-abdominal hemorrhage (black arrows)

And extensive intra-abdominal hemorrhage (Figure 2).

Since the patient had no symptoms and her hemodynamics was not impaired, conservative follow-up was planned. Intermittently, three units of erythrocyte suspension transfusion, IV fluid support and N-acetylcysteine ​​infusion were administered. The patient's antiaggregant treatment was switched from ticagrelor 90 mg twice a day to clopidogrel 75 mg once a day. At discharge, she was prescribed with ASA 81 mg once a day and clopidogrel 75 mg once a day. No bleeding or ischemic event was observed in approximately 12 months of the follow-up. The patient, who was switched to clopidogrel monotherapy in the 12th month, continues to be followed in the cardiology outpatient clinic.

## Discussion

Although there was a decrease in ischemic events with the introduction of potent P2Y12 inhibitors in patients with the ACS, clinicians started to face many bleeding complications in different patient groups in clinical practice. For this reason, the benefit seen in terms of antiaggregant activity especially in the early period has begun to decrease due to some other side effects such as bleeding and dyspnea associated with chronic use, and de-escalation of these strong antiaggregant drugs to classical clopidogrel at an appropriate time has become the focus of the studies [6].

In de-escalation strategies, in line with the latest expert consensus report, two different approaches are recommended with platelet function tests (PFT) and based on a genetic basis [7]. However, rather than relying on any test in clinical practice, for reasons such as drug-related side effects (dyspnea, arrhythmia, etc.), major and minor bleeding complications, newly started anticoagulant drugs, drug incompatibility or emerging clinical conditions such as previous stroke; de-escalation of potent antiaggregant to classical clopidogrel appears to be applied [8]. As a matter of fact, in our patient, de-escalation was applied to clopidogrel due to the major bleeding complication in hospitalization without any PFT or genetic testing. In this case, it becomes a matter of discussion whether there will be an increase in ischemic events and adverse cardiac events due to the transition to early clopidogrel, especially in elderly patients. In the tropical ACS randomized trial with prasugrel, another potent P2Y12 inhibitor, PFT-based de-escalation to clopidogrel was found to be non-inferior in net clinical benefit, including death, MI and stroke at one-year follow-up compared to standard prasugrel use [9]. In the recent 'a multicenter trial to assess the MIcrovascular integrity and left ventricular function Recovery after clopidogrel or TicagrelOr administration, in patients with STEMI treated with thrombolysis (MIRTOS)' study comparing ticagrelor and clopidogrel in patients with ST-elevation MI patients under 75 years of age treated with thrombolytics, Ticagrelor did not show any superiority to clopidogrel in terms of microvascular functions, and it was shown that there was no difference between the two factors in terms of major adverse cardiac events (MACE) and bleeding complications [10]. According to the Swedish Web-system for Enhancement and Development of Evidence-based care in Heart disease Evaluated According to Recommended Therapies (SWEDEHEART) records, it was reported that at the age of 80 years and above ticagrelor decreased ischemic events and stroke rates compared to clopidogrel, but significantly increased death and bleeding complications. However, it has been emphasized that there is a need for randomized studies, especially in elderly patients [11]. In the light of these data, it would be reasonable to routinely evaluate the application of clopidogrel de-escalation during the in-hospital stay 24 hours after the index event without such adverse events, especially in elderly patients who are prone to complications. Although it is necessary to use an emergency ticagrelor loading due to the rapid antiaggregant effect, we think that a comprehensive, near-term evaluation should be made routinely in terms of clopidogrel de-escalation, considering the clinical features. This major bleeding complication in our case supports this clinical approach.

## Conclusions

Although potent P2Y12 inhibitors reduce ischemic events, they should be used with caution due to bleeding side effects. It should be kept in mind among the possible causes of bleeding in intrahepatic hemorrhages, which are vital in bleeding complications that may develop when using this drug group in ACS treatment. The use of clopidogrel as an antiaggregant seems to be a safe haven, especially in elderly patients. Although it is necessary to use P2Y12 inhibitor due to urgent antiaggregant activity, we believe that the implementation of de-escalation to clopidogrel treatment would be reasonable in the early hospitalization. There is a need for randomized controlled studies on this subject.
